# Seaweed liquid extract AS novel sustainable solutions for phycobioremediation plant germination, and feed additive for marine invertebrate copepod

**DOI:** 10.1038/s41598-024-80389-z

**Published:** 2024-11-28

**Authors:** Mohamed Ashour, Hanan M. Khairy, Ahmed Bakr, Mostafa Matter, Ahmed E. Alprol

**Affiliations:** 1 National Institute of Oceanography and Fisheries (NIOF), Cairo, 11516 Egypt; 2 Environment and Bio-Agriculture Department, Faculty of Agriculture, Al-Azhar University, Cairo, 11884 Egypt

**Keywords:** Adsorption, EDX, FTIR, *Jania rubens*, Plackett–Burman factorial design, *Oithona nana*, *Pterocladia capillacea*, Seaweed extract TAM, SEM, *Ulva lactuca*, Biochemistry, Zoology, Environmental sciences, Chemistry

## Abstract

Several studies have shown the importance of using seaweed liquid extract (True-Algae-Max, TAM) as a fish feed additive, and fish-water conditioner. In addition, TAM has demonstrated significant growth improvement when used as a plant growth biostimulant. This study investigates whether seaweed liquid extract (TAM) can achieve good results in new experimental fields such as chromium remediation, plant germination, and live feed supplementation for marine invertebrate Copepod (*Oithona nana*). In this study, several doses of TAM were tested, for the first time, for their impact on the remediation of chromium (Cr^6+^) ions from aqueous solutions and as an aqua feed additive for marine copepods (*Oithona nana*). In addition, it has been tested as promising for the seed germination of Fenugreek (*Trigonella foenum-graecum*) and Faba bean (*Vicia faba* L.). The most important factors influencing the removal (%) of Cr^6+^, identified using a two-level Plackett–Burman factorial design, were selected for additional optimization utilizing a rotatable central composite design. The maximum adsorption of Cr^6+^ was 93.65% under ideal operating circumstances, which included an initial Cr^6+^ concentration of 60 mg L^−1^, a temperature of 25 °C, a pH of 3, a TAM biomass of 0.05 g, and a contact time of 60 min at agitation conditions. Plackett–Burman design data shows the significance of each factor and how well the model fits the Cr^6+^ removal. The results of the germination experiment revealed that the highest significant increase in seed germination was achieved using a TAM level of 0.30 mg mL^−1^ with *V. faba* (88%) and 0.03 mg mL^−1^ with *T. foenum-graecum* (96.6%). Additionally, compared to the control group, TAM at a level of 0.037 mg mL^−1^ showed high root length enhancement on *V. faba* (184%) and *T. foenum-graecum* (188%). The results of the copepod *O. nana* feeding additive experiment found that the group fed on starch supplemented with TAM at a level of 0.3 mL L^−1^, compared to the control group that fed starch only, showed the highest increment in population growth (134.74%), fecundity (270.16%), and population composition of males (133.45%), adults (120.37%), and nauplius (203.18%). Moreover, compared to the control group, the copepod that fed starch supplemented with TAM levels achieved the highest Omega-9 content. In conclusion, it is shown that TAM is a feasible, efficient, and sustainable solution for biodegradable adsorbent for the Cr^6+^ from aqueous solution, enhances plant seed germination and root length*,* and is a novel feed additive for marine copepod *O. nana*, especially in marine invertebrate hatcheries.

## Introduction

Seaweeds have long been recognized for their ecological and economic significance^1^. This marine organism exhibits an incredible diversity of species and possesses unique biochemical compositions that offer immense potential for various industrial and biological applications^2,3^. Among the many valuable constituents found in seaweeds, liquid extracts derived from them have emerged as a revolutionary sustainable solution with multiple benefits^4^.

Regarding sustainable and modern agriculture, the use of chemical fertilizers in modern agriculture has raised concerns regarding their environmental impact, including soil degradation, water pollution, and disruption of the ecosystem^5^. As a result, there has been a growing demand for sustainable alternatives such as biofertilizers^2,6^. Seaweed liquid extract, derived from various species of macroalgae, has shown great potential as a sustainable biofertilizer due to its rich nutrient content, growth-promoting compounds, and positive effects on soil health^7^. The liquid extract derived from seaweed is a valuable source of macronutrients (including nitrogen, phosphorus, and potassium)^8,9^ and micronutrients (including iron, zinc, manganese, etc.) necessary for plant^9,10^. Additionally, seaweed extract is a valuable source of natural plant growth regulators (including gibberellins, cytokinins, auxins, etc.) which plays a pivotal role in improving overall aspects of plant growth^11^. Kumar and Sahoo^12^ demonstrated that the seaweed extracts have an effective role in helping plants against a wide range of environmental challenges like salinity, extreme temperatures, drought, and deficiencies in crucial elements. Moreover, they have been shown to assist in combating diseases triggered by living organisms.

The enhancement of plant resistance is typically associated with the increased production of proteins and metabolites that inhibit pathogens. These alterations in the biochemical composition can lead to advantageous improvements in the nutritional and overall health quality of crops. Fenugreek (*Trigonella foenum-graecum*) and Faba bean (*Vicia faba* L.) are vital crops for agriculture and nutrition even human and/or economic livelihoods^13^. Fenugreek is prized for its culinary and medicinal uses, while Faba beans are important protein sources^14^. Both crops play crucial roles in sustainable agriculture, biodiversity conservation, and economic livelihoods worldwide^15^. The seed germination of *T. foenum-graecum* and *V. faba* L. is crucial for several reasons^16^. Seed germination is a critical stage in the life cycle of Fenugreek and Faba bean crops, influencing their growth, yield potential, overall health, and economic significance^17^. Successful and healthy germination is a key factor in producing stronger and healthier plants, a high yield, and a higher quantity of quality seeds^18^. Several Reports have discovered that the use of seaweed extracts offers various beneficial effects on crops. These effects encompass early seed germination and establishment, enhanced crop performance and yield, heightened resistance to both biotic and abiotic stressors, and prolonged postharvest shelf life of perishable goods^19–21^. Numerous studies have reported significant improvements in plant growth parameters, including increased shoot and root biomass, enhanced photosynthetic efficiency, improved nutrient uptake, and higher yields, following the application of seaweed liquid extract These positive effects can be attributed to the bioactive compounds present in the extract, which stimulate physiological and biochemical processes in plants, leading to enhanced growth and productivity^22^.

Regarding sustainable biophycoremediation of seaweed liquid extract, it is a promising approach in the field of environmental restoration^23,24^. Seaweed liquid extract is particularly effective in the remediation of pollutants such as heavy metals, dyes, organic compounds, and excess nutrients. Heavy metals can accumulate in soil and water, posing significant risks to ecosystems and human health^25,26^. Seaweed liquid extract aids in the absorption and sequestration of heavy metals, reducing their presence in the environment^27^. The utilization of seaweed liquid extract in biophycoremediation holds great promise for the restoration of contaminated environments^28^. Its nutrient-rich composition and growth-promoting properties contribute to the growth and activity of algae, facilitating the effective removal and transformation of pollutants. By harnessing the potential of seaweed liquid extract, we can work towards creating cleaner and healthier ecosystems. Recently, the seaweed liquid extract (TAM^®^) showed multi-purpose novel solutions such as Phytoremediation, antimicrobial activities, and aquafeed additive for marine shrimp^29,30^. As a novel adsorbent, the adsorption of anionic methyl orange (MO) dyes from an aqueous solution using seaweed liquid extract (TAM®) has been investigated by Alprol et al.^29^. In this study, using TAM®, batch adsorption trials were conducted to assess the impact of various factors, including pH, TAM® sorbent doses, temperature, agitation duration, and initial concentration of MO dye parameters. The highest dye adsorption, reaching 90.34% and 46.09 mg g^–1^, was observed when using 0.4 g of TAM® biomass and 16.88 mg g^−1^ at a pH of 1. They concluded that TAM® biomass serves as a novel and cost-effective substitute adsorbent with the potential for removing MO from water solutions. Moreover, the application of TAM’s methanol extract demonstrates significant antibacterial efficacy, showing activity at concentrations of 500 mg ml^−1^ against *Staphylococcus aureus*, *Salmonella typhimurium*, and *Pseudomonas aeruginosa*, and at 250 mg ml^−1^ against *Escherichia coli*. Generally, chromium Cr(VI) is a highly toxic heavy metal and harms the environment, as well as aquatic and terrestrial organisms^31^. Excessive discharge of these pollutants damages the mucous membranes, skin diseases, and respiratory tract infections, and even cancer in humans when ingested^32^. Therefore, to ensure environmental safety, it is crucial to remove these contaminants from the ecosystem using sustainable methods^33^. While several methods such as coagulation, ion exchange, precipitation chemical, and reduction, are commonly used for Cr(VI) removal from wastewater, most of these methods are impractical and expensive. Moreover, existing sorbents have limitations in terms of their ability to function under natural pH conditions, extended contact time, and low adsorption capacities. As a result, there is an urgent need to develop cost-effective and efficient adsorbents that can operate under natural pH, require less contact time, and have high adsorption capacities^34^. Adsorption is a highly attractive option due to its effectiveness, economic viability, and ease of operation^35^. It is an effective biological treatment method that utilizes low-cost biosorbents to remove toxic heavy metals from wastewater^36^. Adsorption holds great promise as an alternative technique for removing heavy metal ions, offering advantages such as cost-effectiveness, high metal binding ability, high efficiency in diluted effluents, environmental friendliness, and the possibility of regenerating biosorbents and recovering metals^37,38^.

Regarding sustainable feed additives for aquaculture activities, marine aquaculture sustainability faces challenges related to nutrition, disease prevention, and environmental sustainability^39^. In marine hatcheries, live feeds play a crucial role as the primary diet for marine larvae^40^. Copepoda, a group of small crustaceans, plays a crucial role in marine ecosystems as primary consumers and a vital food source for various aquatic organisms. It plays a vital role as trophic linkages within marine ecosystems, connecting primary producers and secondary consumers^41^. They occupy significant positions in pelagic marine food webs, particularly concerning gelatinous zooplankton such as jellyfish, which often prey on copepods^42^. Over 60 copepod species have been successfully cultured under laboratory conditions, and nearly 30 culture methods have been reported^43,44^. Within the copepod order, Cyclopoida stands out as the primary consumer of organic matter and the key transporter of energy to higher trophic levels, including small fish, larvae, and juveniles of aquatic species in the marine ecosystem^45^. The marine Cyclopoida species *Oithona nana* is widely recognized as one of the most successfully cultured Cyclopoida species in marine hatcheries^46^. This species meets the nutritional requirements of marine larvae and serves as mobile carriers of essential nutrients. The cultivation of copepods for aquaculture purposes often relies on the availability of suitable feed sources. According to our best knowledge, no previous work studied the impact of seaweed liquid extract on Cyclopoida species *Oithona nana.* Seaweed liquid extract has emerged as a revolutionary Copepoda feed additive due to its nutritional composition and bioactive compounds. The bioactive compounds present in seaweed liquid extract, such as polysaccharides, also provide immunostimulatory and health-enhancing benefits to copepods, promoting their growth and overall well-being^47^. Seaweeds and or their extract are rich in proteins, essential amino acids, vitamins, minerals, and omega-3 fatty acids, making them excellent dietary supplements for copepods and other aquatic organisms^48^. As previously reported, TAM® contains several biomolecules such as phenolic compounds, fatty acids, polysaccharides, and pigments that have improved the growth, nutrient utilization, immune response, and gene expression of cultured aquatic animals^4,49,50^. On the other hand, TAM® reported to have antimicrobial and antioxidant activities, improve disease resistance, improve water quality parameters, and enhance the overall health conditions of several aquatic animal species, especially Nile tilapia (*Oreochromis niloticus*)^33,34^ and whiteleg shrimp (*Litopenaeus vannamei*)^4^.

Due to the TAM® unique composition, TAM® contains various biomolecules, showing several bioremediation activities, growth, and immunity enhancement of several plants and aquatic animals. Therefore, this comprehensive work aims to shed light on the remarkable applications of seaweed liquid extract, particularly in the realms of sustainable biofertilizers, biophycoremediation, and aquaculture feed supplementations.

## Materials and methods

### TAM^®^ preparation and characterization

TAM®, a liquid extract derived from seaweed, is a patent submitted by Ashour ^51^. Ashour et al. ^52^ previously described the TAM® manufacturing method. In summary, one Chlorophyceae species (*Ulva lactuca*) and two Rhodophyceae species (*Pterocladia capillacea* and *Jania rubens*) were collected from rocky regions around Alexandria. These seaweeds were subsequently purified, cleansed, air-dried, ground into powder, and stored in ambient conditions (22 °C) for subsequent analysis and TAM® preparation. Following the drying phase, the TAM® biomass was dried and sieved to obtain suitable particles (125 μm mesh) using the laboratory sieve. As detailed in our earlier work^52^, the composition of TAM® was examined using a variety of techniques, such as phytochemical, biochemical, chemical, and physical investigations. According to the physical analaysis, TAM® has a dark-brown hue, a density of 1.2, a pH range of 9 to 9.5, and an aroma similar to that of marine seaweed. TAM®'s biochemical makeup revealed that it comprises 2.6% total dissolved solids, 8.2% total organic matter, and 15% total polysaccharides. According to the physical characteristics test, TAM® has a dark-brown tone, a density of 1.2, a pH range of 9–9.5, and a taste similar to that of marine seaweed. TAM®'s biochemical analysis revealed that it involves total dissolved solids, total organic matter, and total polysaccharides of 2.6%, 8.2%, and 15%, respectively. Additionally, phytochemical tests revealed that TAM® has a DPPH inhibitory activities of 70.33% . Moreover, TAM®revealed a significant amount of total flavonoid compounds, total antioxidant capacity, total phenolic compounds, and total ascorbic acid (2.60 mg g^−1^, 54.52 mg g^−1^ (% DM), 101.67 mg g^−1^, and 1.66 mg g^−1^). According to our earlier work^52^, the biologically active material profile of TAM® revealed nine significant phytochemical compounds with growth-promoting, immunity-boosting, antioxidant, and antibacterial properties based on a GS-Mass analysis^50^, as displayed in Supplementary Fig. 1.

#### TAM FTIR analysis

Fourier Transform Infrared Spectroscopy analysis (FTIR) was used to confirm the presence of functional groups in the dry TAM® biomass samples. The TAM® biomass samples were incorporated with KBr pellets and the FTIR spectra were measured using a Thermo Fisher Nicolete IS10, USA spectrophotometer within the range of 400–4000 cm^−1^.

#### Scanning electron microscopy (SEM) and energy dispersive X-ray (EDX)

SEM and EDX were used to verify the morphological differences and to determine the elemental composition at the dry TAM® biomass samples to examine the algal cell surfaces and to evaluate the Cr^+6^ adsorption. The samples were coated with gold and were examined at different magnifications at 20 kV.

### Germination experiment

#### Seed germination procedures

The Faba bean cv. Giza 716 (*Vicia faba* L.) and Fenugreek (*Trigonella foenum-graecum*) were obtained from the Legume Research Department, the Field Crop Institute, Agricultural Research Center, Ciro, Egypt. Seeds were identified and the germination rate (%) experiment of *V. faba* and *T. foenum-graecum* was conducted at the Environment and Bio-Agriculture Department, the Faculty of Agriculture, Al-Azhar University, Ciro, Egypt.To prepare the plant seeds, equal-sized samples were surface sterilized by immersing them in a 10% sodium hypochlorite solution (NaClO) for 10 min. Subsequently, the seeds were soaked in distilled water for 24 h at 25 °C. Then, the seeds were allowed to germinate in sterilized Petri dishes. Each Petri dish contained one piece of filter paper and 5 mL of different concentrations of crude TAM® (0, 0.037, 0.075, 0.150, 0.30, 0.60, 1.20, 2.40, and 4.80 mg mL^−1^) dissolved in distilled water, three replicates/concentration. 10 seeds were transferred and placed in each dish to be germinating. The distance between each seed was 1 cm. Each treatment has three replicates and distilled water was used as the control for the untreated experiments.

#### Testing parameters

To determine the germination rate and root growth, the seeds’ germination was investigated for one week. The percentage of seed germination rate (%) and the length of seedling roots (cm) were then calculated and measured, respectively. Each treatment was replicated three times. The following formula was used to calculate the percentage of seed germination for both treated and control treatment:

1 $${\text{Germination}}\left( \% \right) = {\text{Number of germinated seeds}}/{\text{Number of used seeds}}) \times {1}00$$

### Aquaculture feed additive experiment

#### Copepod *Oithona nana* isolation

The marine copepod individuals were isolated from an aquaculture earthen pond located at El-Max Research Station, following the Alexandria Branch of the National Institute of Oceanography & Fisheries, (NIOF), Egypt. The collection period of the copepod was performed during the spring of 2023. The water quality of the earthen pond was recorded at noon. The temperature, salinity, and pH were 22.5 ± 1.5 °C, 31 ± 1 ppt, and 7.57, respectively. Water samples collected for copepod isolation were collected following the protocol described previously^53^. A binocular stereomicroscope (Optika Microscopes, B190/B-290, Ponteranica, Italy) was used for the initial examination, copepod isolation, and morphological investigation. The individual identification and taxonomic characterization were performed by the Hydrobiology Lab., Alexandria Branch, Marine Environment Division of NIOF. After morphological classification, the identified adult individuals were characterized as Cyclopoida: *Oithona nana*.

#### Experimental regime and design

For 15-days of acclimatization, the *O. nana* individuals were maintained and subcultured under laboratory-controlled conditions of temperature (27 ± 1 ^°^C), salinity (21 ± 1 ppt), pH (7.7 ± 0.15), and continuous gentle aeration. Moreover, during the 15-day acclimatization period, *O. nana* individuals were fed only corn starch, with a concentration of 1 g 10^−6^ individuals, to ensure that the digestive tract of *O. nana* was completely emptied from any other food before the beginning of the experiment. After 15 days of acclimatization period, the isolated *O. nana* individuals were divided into five groups and stocked into plastic containers filled with 5 L culture water, each group had three replicates. The stocking density of *O. nana* in each group was estimated to be 1 individual (Ind. mL^−1^). Therefore, the initial population number of each plastic container was 5000 Ind. L^−1^. The experiment was continued for 21 days. Based on our primary and laboratory experimental trials, five groups were performed in this experiment; the first group (CS) was the copepod that fed on the stock solution of corn starch with a ratio of 1 g 10^6^ Ind., as a control experimental diet. Groups from 2 to 5 (S-TAM_1_, S-TAM_2_, S-TAM_3_, and S-TAM_4_) were the copepods that fed on the stock solution of corn starch, with the same ratio of CS, supplemented with several levels of crude TAM® (0.1, 0.2, 0.3, and 0.4 mL L^−1^ of water culture, respectively). The experimental culture conditions were maintained under laboratory-controlled conditions of temperature (27 ± 1 °C), salinity (21 ± 1 ppt), pH (7.7 ± 0.15), and continuous gentle aeration.

#### Tested parameters

##### Morphological tested indicators

Population growth (increase in number), growth rate (r), population composition (number of males, females, adults, and nauplius), and fecundity (eggs/female) have been investigated. To investigate the population growth of *O. nana,* during the experiment period (day 5, day 10, day 15, and day 21), from every replicate of each group, 20 mL of culture water was taken to estimate the increase in the number of *O. nana* (ind. mL^*−*1^). The population growth rate (r) of *O. nana* was calculated based on the equation reported by Yin et al. ^54^:

2 $${\text{r}} = \left( {{\text{lnN}}_{{\text{e}}} - {\text{lnN}}_{{\text{i}}} } \right)/{\text{t}}$$

where N_i_ and N_e_ are the initial and final population numbers, while t is the incubation time in days.

To estimate the population composition (number of males, females, adults, and nauplius), at the end of the experiment, from each replicate in all experiment groups, about 100 individuals of *O. nana* were harvested by using a plankton net (38 µm). The harvested individuals were fixed in a 4% formalin stock solution and investigated under a microscope (Optika Microscopes, B190/B-290, Ponteranica, Italy). To investigate the fecundity (number of eggs/female), a total of 25 healthy-carrying females were selected from each replicate and carefully placed on a Petri dish for examination.

##### Fatty acid profile analysis

To estimate the fatty acids profile of *O. nana* fed different diets, after 21 days, from all replicates, all individuals were harvested and preserved at − 80 ^°^C. The extraction of fatty acids was conducted, and the profiles of these fatty acids were determined following the methodology described in the study conducted by Zaki et al. ^55^. For the fatty acids profile, the Hypocholesterolemic/Hypercholesterolemic ratio (HHR) was assessed by applying the formula previously described by Chen and Liu ^56^, as follows:

3 $${\text{HHR}} = \left( {{\text{cis - C18:1}} + \sum {\text{PUFA}}} \right)/\left( {{\text{C12:}}0 + {\text{C14:}}0 + {\text{C16:}}0} \right)$$

### Bioremediation experiment

#### Preparation of Cr^6+^ solution

All used chemicals were of analytical grade, and the adsorption experiments were carried out at room temperature (25 ± 2 °C). A 1000 ppm stock solution of Cr^6+^ was prepared by dissolving 2.834 g of K_2_Cr_2_O_7_ in 1000 mL of double distilled water in a measuring flask. Before performing the batch experiments, the initial pH of each chromium concentration solution was adjusted at pH 1.5 with 0.1M HCl or 0.1M NaOH. The concentrations of free Cr^+6^ ions in the stock solution and unabsorbed chromium ions in the adsorption medium were determined by spectrophotometer at λ = 540 nm using 100 ppm diphenyl carbazide, which gives a red-violet colored complex. After the adsorption process is complete, the TAM® biomass should be separated from the chromium-containing solution. This can be achieved using filtration methods. The specific method used should be stated explicitly, the biomass was separated from the Cr^6+^ solution by filtration using Whatman filter paper. Then the filtrate was analyzed for metal content using spectrophotometry techniques with diphenylcarbazide as the reagent at the maximum wavelength of 540 nm^57^.

#### Selection of significant variables for Cr^+6^ removal by Plackett–Burman design

The Plackett–Burman Design (PBD), is an efficient screening method to detect the significant variables among the large number of variables that influence a process^58^. PBD was used for the selection of the variables that had a significant effect, either positively or negatively on Cr^+6^ adsorption out of six variables. The six variables (independent variables) included: different contact times (60 and 120 min), Cr^+6^ ions concentration (30 and 60 mg L^−1^), and two different initial pH levels (3 and 5) which were adjusted with 0.1 N HCl and 0.1 N NaOH, temperature (25 and 35 °C), biomass doss (0.05 and 0.1 g) and static or agitation condition. Each variable was examined in two levels, low (−) and high (+) level. 12 runs Plackett–Burman design were used to evaluate the effect of the selected six variables on the Cr^+6^ removal efficiency. In the experimental design, each row represents an experiment and each column represents an independent variable (Table 1). The dry biomass of the adsorbent was thoroughly mixed with the solution of Cr^+6^ in Erlenmeyer flasks. The suspensions were kept static or with agitation for a specific contact time at the selected temperature. Plackett–Burman experimental design is based on the first-order model equation^59^:

4 $$Y = \beta 0 + \Sigma \beta iXi$$

where Y_i_ is the measured response (Cr^+6^ removal %), β0 is the model intercept, *β*_*i*_ is the linear coefficient, and X_i_ is the level of the independent variable.

**Table 1:** Twelve attempts using the coded and actual amounts of independent variables in conjunction with the observed values of Cr^+6^ removal by TAM biomass.

Run number	Coded and actual levels of independent variables											
	Contact time (min)		Cr^6+^ concentration (mg L^−1^)		pH		Temperature (°C)		Dose (g)		Static-agitation	
1	− 1	60	− 1	30	− 1	3	1	35	1	0.1	1	Agitation
2	− 1	60	− 1	30	1	5	1	35	1	0.1	− 1	Static
3	− 1	60	1	60	1	5	1	35	− 1	0.05	1	Agitation
4	1	120	− 1	30	1	5	1	35	− 1	0.05	1	Agitation
5	1	120	− 1	30	− 1	3	− 1	25	1	0.1	1	Agitation
6	− 1	60	1	60	− 1	3	− 1	25	− 1	0.05	1	Agitation
7	1	120	1	60	− 1	3	1	35	− 1	0.05	− 1	Static
8	1	120	1	60	1	5	− 1	25	1	0.1	1	Agitation
9	− 1	60	− 1	30	− 1	3	− 1	25	− 1	0.05	− 1	Static
10	1	120	− 1	30	1	5	− 1	25	− 1	0.05	− 1	Static
11	1	120	1	60	− 1	3	1	35	1	0.1	− 1	Static
12	− 1	60	1	60	1	5	− 1	25	1	0.1	− 1	Static

#### Optimization of Cr^+6^ removal

Based on the results of PBD, a three-factor, five-level rotatable central composite design was performed to determine the optimum levels of the significant variables and the individual interactions between the selected variables with high influence on Cr^+6^ removal. Linear, quadratic, and interaction effects of the three variables on Cr^+6^ removal were calculated. The relationship between the Cr^+6^ removal (Y) viz the significant independent variables (X_1_, X_2,_ and X_3_) is given using the following second-order polynomial equation ^59^:

5 $${\text{Y}} = \beta_{0} + \Sigma \beta_{{\text{i}}} {\text{X}}_{{\text{i}}} + \Sigma \beta_{{{\text{ii}}}} {\text{X}}_{{\text{i}}}^{{2}} + \Sigma \beta_{{{\text{ij}}}} {\text{X}}_{{\text{i}}} {\text{X}}_{{\text{j}}}$$

In which Y is the predicted Cr^+6^ removal, β0 is the regression coefficients, βi is the linear coefficient, βii is the quadratic coefficients, βij is the interaction coefficients, and Xi is the coded levels of independent variables.

### Data statistical analysis

Each assay was performed in three independent replicates (± standard division). Before statistical analysis, Levene’s test was employed to confirm the normality and homogeneity assumptions, and the results (%) were arc-sin transformed^60^. The statistical analysis was conducted using the SPSS Statistics Software, which involved performing one-way ANOVA followed by the Duncan ^61^ test at a significant level of *p* ≤ 0.05. Finally, the figures were created using Graph Pad (Prism 8) Statistics Software^62^. Minitab version 19 for Windows software was used for the experimental designs and statistical analysis. The response surface and contour plots were used to assess the relationship between the significant variables. The amount of Cr^+6^ ions absorbed per gram (mg g^–1^) of TAM ®biomass may be determined by using the following equation^36^ at equilibrium:

6 $${\text{q}}_{{\text{e}}} = \left[ {\left( {\left( {{\text{C}}_{{\text{i}}} - {\text{C}}_{{\text{e}}} } \right) \times {\text{V}}} \right)/{\text{M}}} \right]$$

The percentage of Cr^+6^ ions removed (efficiency) obtained by the following equation can also be used to show Cr^+6^ uptake:

7 $${\text{Adsorption }}\left( \% \right) = \left[ {\left( {{\text{C}}_{{\text{i}}} {-}{\text{C}}_{{\text{e}}} } \right)/{\text{C}}_{{\text{i}}} } \right] \times {1}00$$

where: C_i_ is the initial concentration of Cr^+6^ ions and C_e_ is the equilibrium concentration of Cr^+6^ ions (mg L^─1^), m (g) weight of TAM®, and V (L) volume of the Cr^+6^ ions solution, respectively.

### Ethical approval

All mthods in this work were approved performed in accordance with the relevant guidelines and regulations by the National Institute of Oceanography and Fisheries (NIOF) Committee for Institutional Care of Aquatic Organisms and Experimental Animals (NIOF- IACUC, Code: NIOF-AQ3-I-24-R-028).

## Results and discussion

### *TAM*® *characterizations*

**Fig. 1:**
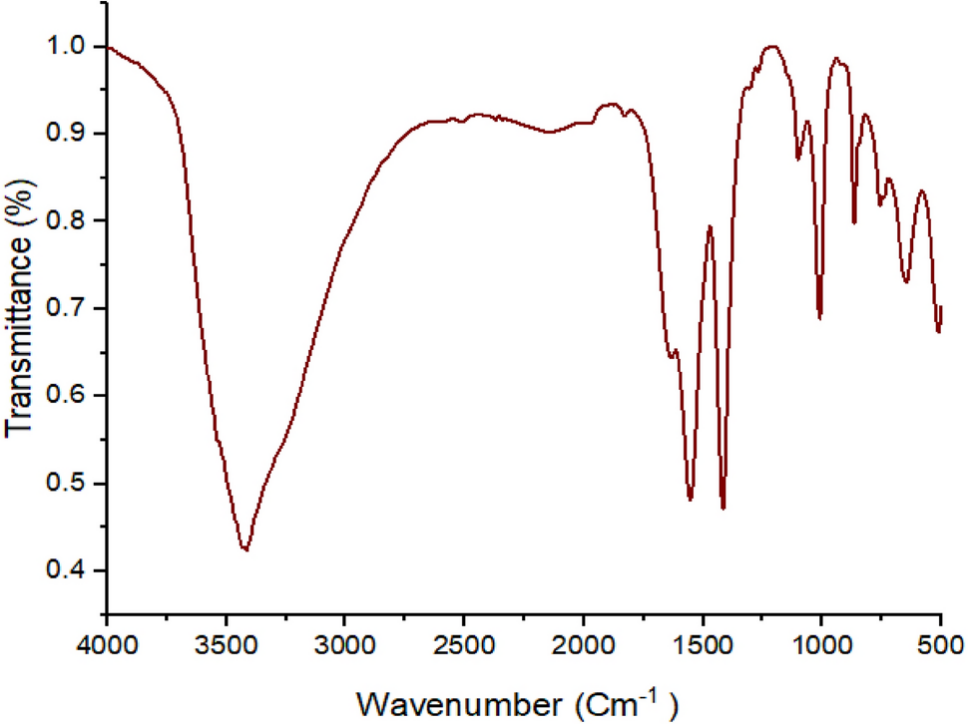
Functional groups and matching infrared frequencies in TAM adsorbent.

**Fig. 2:**
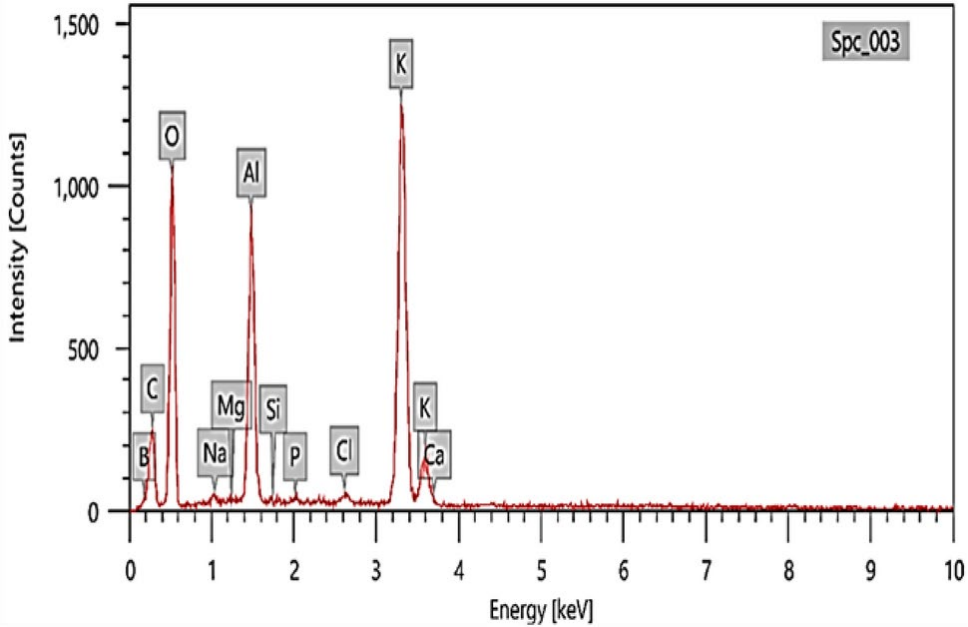
EDX studies for TAM.

FTIR spectroscopy is useful in determining the type of bonds that are present and in identifying functional sites on the cell surface. Figure 1 displays the FTIR spectra of the sample and the organic functional groups and matching infrared frequencies found in TAM® and the biomasses utilized in this investigation. The band that lies at around 3416 cm^−1^ in the spectra shows that the True-Algae-Max under study includes hydroxyl and amine groups^63^. The C-N stretching vibration was identified as the source of the band at 2138 cm^−1^. The spectra display the distinctive C=C vibration bands at 1634 and 1552 cm^−1^. The vibrational band observed at 1829.34 cm^−1^ was the result of the C=O groups of anhydrides. The bands that show up in the 1390–1515 cm^−1^ range can be attributed to ν(C=C) and v(C=N). The bending vibration of the adsorbed water and the carbonyl groups (C=O) indicating the presence of phenols, carboxyl acids, and aldehydes were attributed to the spectral bands that were found at 1675 and 1609 cm^−1^. The C–O–C group and Si–O stretching vibration of the adsorbent are represented by the bands at 1120–1000 cm^−1^. In the amide group, the s peaks at 1423 and 1377 cm^−1^ were attributed to CH_3_. The peak was attributed to C-H stretching at 865 and 756 cm^−1^. The C–S linkage and halogen of the material (Bromo-compounds groups) were attributed to the peak at 647 cm^−1^, while the Se–O–Se and Cu–O stretching vibrations were indicated by the peaks at 511.7 and 471.5 cm^−1^, respectively. Elemental analysis on regions as small as nanometers in diameter can be obtained using EDX in conjunction with these imaging techniques. X-rays that are typical of the elements existing on the sample are produced when the electron beam impacts it. The EDX analysis can be performed to identify the elemental composition of individual spots or to map out the lateral distribution of the components from the photographed area^64^. The EDX analyses showing the elemental composition are presented in Fig. 2. The elemental compositions of the True-Algae-Max used in this study are given in Table 2 and have higher oxygen and carbon percentages at 57.3 and 21.1%, respectively. In addition, a higher boron percentage (3.64%) is due to the presence of sulfated polysaccharides in the adsorbent structure. With the highest mass percentage (50.34%) and atomic percentage (57.30%), oxygen is the most abundant element in the TAM® material. This is expected as oxygen is a primary component of organic molecules such as carbohydrates, proteins, and lipids, which are prevalent in algae.

**Table 2:** Element composition percentage of TAM adsorbent.

Element	Mass%	Atom%
B	2.16 ± 0.11	3.64 ± 0.18
C	13.97 ± 0.20	21.18 ± 0.31
O	50.34 ± 0.59	57.30 ± 0.68
Na	0.53 ± 0.06	0.42 ± 0.05
Al	9.72 ± 0.17	6.56 ± 0.11
Si	0.07 ± 0.03	0.04 ± 0.02
P	0.28 ± 0.04	0.17 ± 0.02
Cl	0.40 ± 0.04	0.20 ± 0.02
K	22.37 ± 0.26	10.42 ± 0.12
Ca	0.17 ± 0.05	0.08 ± 0.02
Total	100	100

Potassium is the second most abundant element, with a mass percentage of 22.37% and an atomic percentage of 10.42%. Potassium plays essential roles in various physiological processes in algae, including osmoregulation, enzyme activation, and nutrient uptake. Also, carbon constitutes 13.97% of the mass and 21.18% of the atoms in the TAM® material. As the backbone of organic molecules, carbon’s presence is consistent with the algal origin of the material. Correspondingly, aluminum is present at 9.72% by mass and 6.56% by atom. The source of aluminum could be from the natural environment of the algae, or it may have been introduced during the preparation process. The high percentages of oxygen and carbon, along with the presence of other elements like potassium, phosphorus, and calcium, confirm the organic nature of the TAM® material and its origin from algal biomass. The presence of oxygen-containing functional groups (e.g., hydroxyl, carboxyl) and the overall complex surface chemistry suggested by the elemental composition indicate the potential of the TAM® material for adsorption applications. These functional groups can interact with dye molecules through various mechanisms, such as electrostatic attraction, hydrogen bonding, and van der Waals forces^65^. The high percentages of oxygen and carbon, coupled with the presence of essential elements like potassium, indicate the organic nature of TAM® biomass and its algal origin. The overall elemental profile suggests that the material is rich in organic compounds and contains various elements essential for algal growth and biological activity. The presence of elements like oxygen, phosphorus, and carbon, in the TAM® biomass structure, likely contributes to the adsorbent’s ability to bind and remove heavy metals from aqueous solutions, as shown in the paper’s bioremediation experiments. These elements might provide bonding sites or participate in chelating processes with the heavy metal ions^66^. Also, the presence of potassium and other elements is significant for plant growth as a biofertilizer. In addition, the presence of elements like phosphorus, and carbon, indicates that TAM® is likely to contain important nutrients needed by copepods^67^.

**Fig. 3:**
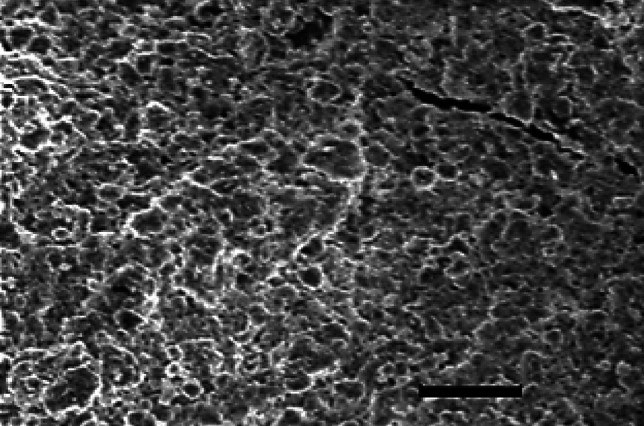
SEM of TAM (10.0 mm-Magnification × 400).

Nevertheless, scanning electron microscopy (SEM) is a powerful technique used to characterize the surface morphology and elemental composition of materials. The SEM image reveals a complex and heterogeneous surface texture (Fig. 3). The SEM image reveals a complex and heterogeneous surface texture. TAM® appears to have a rough, uneven topography with numerous protrusions, cavities, and channels of varying sizes. This irregular and textured surface morphology is characteristic of biological materials and likely results from the combination of different algal species (Rhodophyceae and Chlorophyceae) used to create the extract^68^. The presence of these protrusions, cavities, and channels is highly significant in the context of adsorption processes. A material with a large surface area-to-volume ratio, like TAM®, can provide more binding sites for adsorbates. This irregular structure creates many opportunities for interaction and attachment, potentially enhancing the efficiency of the adsorption process. The image also suggests a possible porous structure within the TAM® biomass^65^. The channels and cavities could serve as “trapping zones” for adsorbate molecules, leading to greater capture and retention during the removal process. This intricate surface morphology is likely due to the combination of the three different algal species, each contributing unique structural features^36^. The presence of these irregularities and the high surface area-to-volume ratio suggest that the True-Algae-Max material may be well-suited for adsorption applications.

### Seed germination

**Fig. 4:**
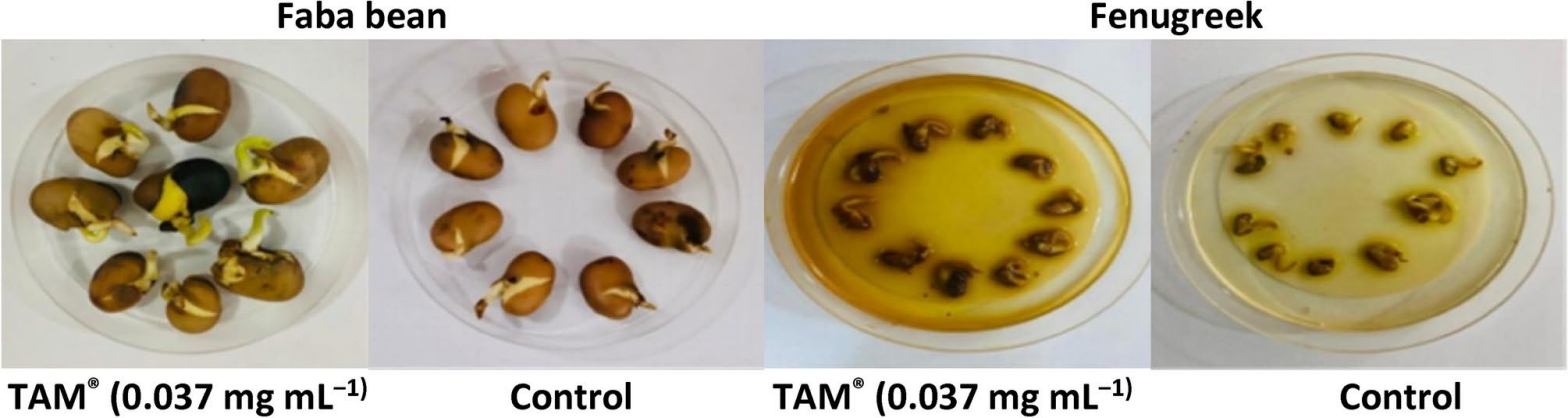
Effect of different concentrations of TAM on faba bean *V. faba* seed root length and germination rate. (**a**) Seeds in the petri dish with control, TAM. (**b**) The effect of different concentrations of TAM.

Efforts to promote optimal germination conditions can contribute significantly to the success of cultivation practices for these important crops^18^. Figure 4 shows the effect of different TAM® concentrations on faba bean *V. faba* and Fenugreek *T. foenum-graecum* seed root length and germination rate.

**Fig. 5:**
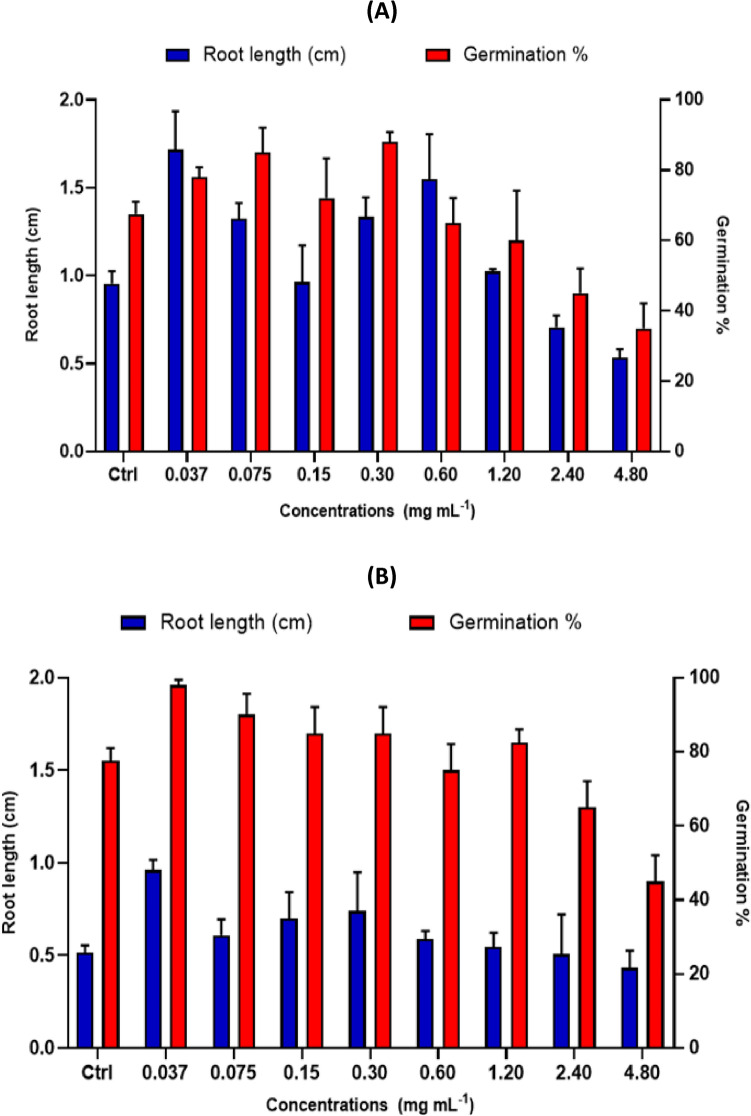
Mean ± SD of seed root length (cm) and germination rates (%) of *Vicia faba* (**a**) and *Trigonella foenum-graecum* (**b**) (n = 3) in response to different concentrations of TAM, compared to control (Ctrl).

The germination experiment showed a significant (*p* < 0.01) increase in seed germination of *V. faba* in all TAM® levels. Compared to the control group, the TAM® groups of 0.30, 0.075, 0.037, and 0.15 mg mL^−1^ significantly enhanced the germination rate of *V. faba* by 88, 85, 78, and 72%, respectively (Fig. 5).

While with Fenugreek *T. foenum-graecum*, the increase in germination rate was only at groups of 0.037, 0.075, 0.15, and 0.30 mg mL^−1^, it was 96.6, 90, 85, and 85%, respectively (Fig. 5). Furthermore, TAM® showed the highest enhanced root length of *V. faba* seeds recorded at 0.037 mg mL^−1^ of 184%, while increasing the root length of *T. foenum-graecum* by 188% when compared to the control. While TAM® concentrations of 0.60 and 1.20 mg mL^−1^ showed almost similar effects to control. The high concentrations of 2.4 and 4.8 mg mL^−1^ did not show a positive effect on seed germination.

Our findings were confirmed by numerous studies that indicated an increase in plant growth parameters, seed germination, vigor, nutrient uptake, and protection against abiotic and biotic stresses when utilizing seaweed extract. This improvement is attributed to the activation of secondary metabolites and the management of defense pathways. As reported previously^19^, TAM® showed 15% of total polysaccharides, besides the several phytochemicals, including phenolic compounds (101.67 mg g^−1^), flavonoid compounds (2.60 mg g^−1^), and ascorbic acid (1.66 mg g^−1^), total antioxidant capacity (54.52 mg g^−1^), and a DPPH inhibition of 70.33%. Besides, as presented in Supplementary Fig. 1, TAM® unveiled several novel bioactive compounds including (a) 5-Silaspiro[4.4]nona-1,3,6,8-tetraene,3,8-bis(diethylboryl)-2,7-diethyl-1,4,6,9-tetraphenyl-, (b) nonadecane, (c) rhodopin, (d) milbemycin B, (e) tridecanoic acid methyl ester, (f) oleic acid, (g) γ-linolenic acid methyl ester, (h) 9,12-octadecadienoic acid, methyl ester, (E, E)-, and (i) phytol. Interestingly, TAM® introduced novel bioactive compounds not previously documented in seaweed extracts, utilized in this study for the first time for plant seed germination. These compounds, including milbemycin-oxime and 5-silaspiro[4.4]nona-1,3,6,8-tetraene,3,8-bis(diethylboryl)-2,7-diethyl-1,4,6,9-tetraphenyl^19,69^. 5-silaspiro[4.4]nona- represents a phyto-bioactive compound with two boron atoms linked to a silicon atom. Boron is essential for plant embryonic growth and metabolism. Further studies and in-depth investigations are still needed on this interesting point.

Our results matched with the work conducted by Mamede et al. ^70^ who concluded that compared to synthetic commercial fertilizers, seaweed polysaccharides have demonstrated remarkable efficacy in enhancing plant growth parameters^71^. Whether applied directly to the soil or sprayed on the foliage, the utilization of seaweed poly- and oligosaccharides can lead to improved seed germination, enhanced plant vigor, increased absorption of soil nutrients, and protection against various abiotic and biotic stresses. These beneficial effects are achieved through the stimulation of secondary metabolite production and the regulation of defense pathways within the plant^72^.

The positive effects of seaweed extract on seed germination and plant growth have been demonstrated across all stages of the plant cycle. Seaweed products have been shown to enhance germination rates and significantly boost seedling vigor by improving root length and density. Recent studies have highlighted the application of aqueous seaweed extracts (*Ulva fasciata, Padina gymnospora,* and *Gracilaria edulis*) as biofertilizers for promoting *Capsicum annuum* seed germination^20^. Additionally, Rengasamy et al.^73^ found that the application of eckol and phloroglucinol, extracted from *Ecklonia maxima*, increased maize seed germination rates by stimulating the enzyme α-amylase, which aids in converting starch into simple sugars essential for maize seedling root metabolism. Seaweed liquid extracts derived from several seaweed species (*U. lactuca, C. sertularioides, P. gymnospora,* and* S. liebmannii*) have been found to enhance germination responses and improve physiological traits in tomatoes. Hernández-Herrera et al.^74^ demonstrated that soil drench application of seaweed extracts was more effective compared to chemical foliar spray. Ahmed et al.^75^ observed that applying *U. fasciata* and *S. lacerifolium* to the soil increased germination rates, and enhanced morphological and biochemical characteristics of radish *Raphanus sativus* L.

### Copepod feeding

Several studies have reported the positive effects of seaweed liquid extract as a feed additive on growth performance, immune response, and gut health of various aquatic species such^76^ as Nile^77^ , red tilapia^78^, shrimp^79^, etc. Seaweed liquid extract has emerged as a valuable feed additive for aquaculture due to its nutritional composition and bioactive compounds. Seaweed extracts are rich in proteins, essential amino acids, vitamins, minerals, and omega-3 fatty acids, making them excellent dietary supplements for aquatic animals^80^. Additionally, seaweed bioactive compounds, such as polysaccharides, have shown immunostimulatory and antioxidant properties, which can enhance the health and disease resistance of aquaculture species^79^. In the current study, the copepod species *O. nana* was selected as an aquatic animal model, due to its important role in marine hatcheries. According to our best knowledge, this is the first report conducted to investigate the impact of seaweed liquid extract as a feed additive for copepod *O. nana*. Table 3 shows the population growth, population composition, and fecundity of copepod *O. nana* feed starch supplemented with different levels of TAM® (0.1, 0.2, 0.3, and 0.4 mL 1 L^−1^ of water culture). Table 3 shows that all *O. nana* groups fed starch supplemented with different levels of TAM® showed significant (p < 0.05) improvement in population growth, population composition (males, adults, and nauplius), and fecundity. The copepod group fed on starch supplemented with TAM® at a level of 0.3 mL 1 L^−1^ of water culture (S-TAM_2_) showed the highest increment in population growth (134.74%), fecundity (270.16%), and population composition of males (133.45%), adults (120.37%), and nauplius (203.18%). These findings may be due to the TAM’s polysaccharides content which previously showed growth-promoter, antioxidant activities, and immunity-enhancer of Nile tilapia^77^.

**Table 3:** Population growth, population composition (males, females, adults, and nauplius), and fecundity of copepod *O. nana* feed starch supplemented with different levels of TAM.

Groups*	Population growth (Ind. L^−1^)	Population composition (Ind. L^−1^)				Fecundity (eggs/female)
		Males	Females	Adults	Nauplius	
CS	6155.26 ± 63.07^d^ (100.00%)	3043.67 ± 24.01^b^ (100.00%)	2043.67 ± 24.01 (100.00%)	5087.33 ± 48.01^b^ (100.00%)	1068.00 ± 15.10^c^ (100.00%)	1.24 ± 0.05^d^ (100.00%)
S-TAM_1_	6642.00 ± 46.86^c^ (107.90%)	3051.33 ± 22.01^b^ (100.25%)	2051.33 ± 22.01 (100.37%)	5102.67 ± 44.02^b^ (100.30%)	1539.35 ± 25.32^b^ (144.13%)	2.75 ± 0.18^b^ (221.77%)
S-TAM_2_	8294.00 ± 91.80^a^ (134.74%)	4062.00 ± 25.63^a^ (133.45%)	2062.00 ± 25.63 (100.89%)	6124.00 ± 51.26^a^ (120.37%)	2170.00 ± 43.59^a^ (203.18%)	3.35 ± 0.10^a^ (270.16%)
S-TAM_3_	7680.33 ± 56.87^b^ (124.77%)	4055.37 ± 33.02^a^ (133.23%)	2055.33 ± 33.02 (100.57%)	6110.69 ± 66.04^a^ (120.11%)	1569.67 ± 31.53^b^ (146.97%)	2.74 ± 0.19^b^ (220.96%)
S-TAM_4_	6207.67 ± 25.58^d^ (100.85%)	3056.30 ± 22.90^b^ (100.41%)	2056.27 ± 22.90 (100.61%)	5112.67 ± 45.80^b^ (100.49%)	1095.00 ± 23.64^c^ (102.52%)	2.32 ± 0.09^c^ (187.10%)

*CS: Copepods fed on corn starch (1 g 10^6^ Individuals) as a control experimental diet. S-TAM_1_, S-TAM_2_, S-TAM_3_, and S-TAM_4_: Copepods fed on corn starch (at a rito of 1 g 10^6^ Individuals) supplemented with several levels of crude TAM (0.1, 0.2, 0.3, and 0.4 mL 1 L^−1^ of water culture, respectively). The presented data are Means ± SD (n = 3). Different letters in the same column are significantly different (p < 0.05). The absence of letters in the same row means no significant differences. The data in brackets means the percentage increase between the TAM supplementation groups (S-TAM_1_, S-TAM_2_, S-TAM_3_, and S-TAM_4_) compared to the control treatment (CS).

Several studies have reported the positive effects of seaweed liquid extract as a feed additive on growth performance, immune response, and gut health of various aquatic species such as Nile tilapia^76^, red tilapia^78^, shrimp^79^, etc. Seaweed liquid extract has emerged as a valuable feed additive for aquaculture due to its nutritional composition and bioactive compounds. Seaweed extracts are rich in proteins, essential amino acids, vitamins, minerals, and omega-3 fatty acids, making them excellent dietary supplements for aquatic animals^80^. Additionally, seaweed bioactive compounds, such as polysaccharides, have shown immunostimulatory and antioxidant properties, which can enhance the health and disease resistance of aquaculture species^79^. In the current study, the copepod species *O. nana* was selected as an aquatic animal model, due to its important role in marine hatcheries. According to our best knowledge, this is the first report conducted to investigate the impact of seaweed liquid extract as a feed additive for copepod *O. nana*. Table 3 shows the population growth, population composition, and fecundity of copepod *O. nana* feed starch supplemented with different levels of TAM® (0.1, 0.2, 0.3, and 0.4 mL 1 L^−1^ of water culture).

**Fig. 6:**
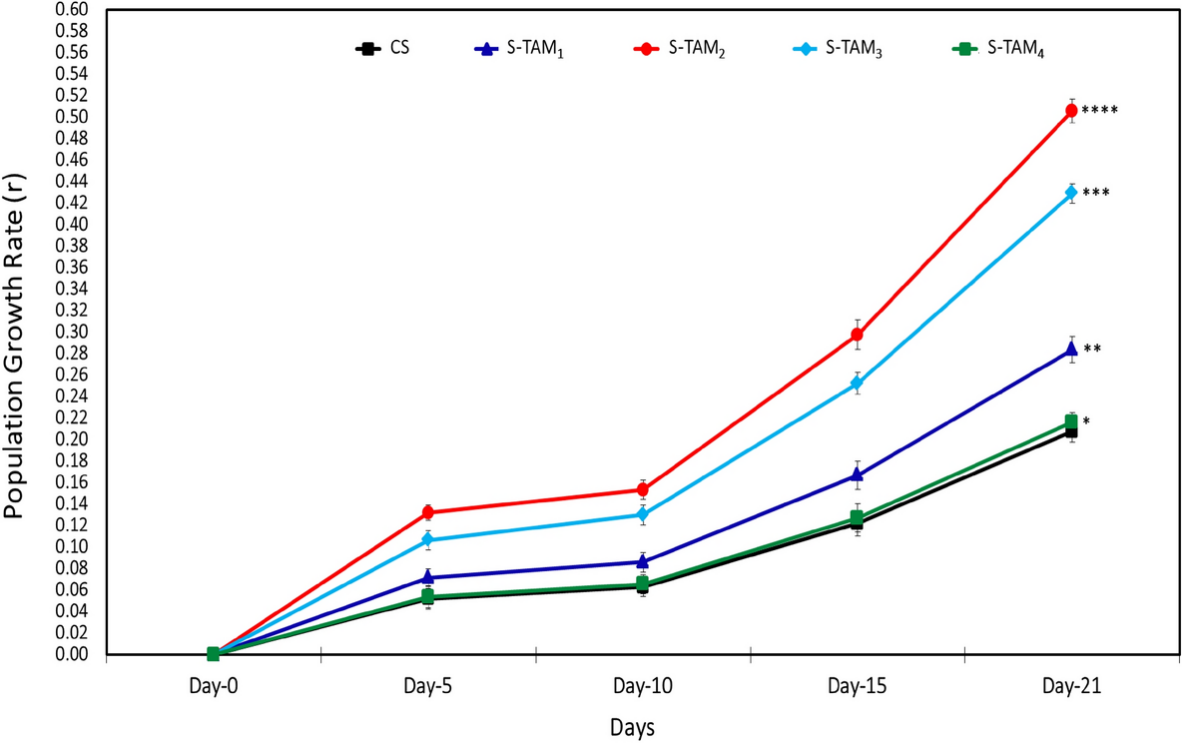
Effect of different crude TAM supplementations on the population growth rate (*r*) of copepods *O. nana*. CS: Copepods fed on corn starch (1 g 10^–6^ Individuals) as a control experimental diet. S-TAM_1_, S-TAM_2_, S-TAM_3_, and S-TAM_4_: Copepods fed on corn starch ( at a rito of 1 g 10^–6^ Individuals) supplemented with several levels of crude TAM® (0.1, 0.2, 0.3, and 0.4 mL L^−1^ of water culture, respectively). The presented data are Means ± SD (n = 3). The stars above each line (**** > *** > ** > *) indicate significant differences (*p* ≤ 0.05) among different diets on the same day.

Figure 6 shows the effect of different crude TAM® supplementations on the population growth rate (*r*) of copepods *O. nana*, compared to the control group (CS). During 21 days of the experiment, the S-TAM_2_ group showed the highest significant (*p* ≤ 0.05) population growth (r = 0.51), followed by S-TAM_3_ (r = 0.43), S-TAM_1_ (r = 0.28), while the lowest significant (*p* ≤ 0.05) population growth was recorded by groups S-TAM_5_ (r = 0.22) and the control group CS (r = 0.21). This finding confirmed that the optimal dose of TAM® is limited to the group of S-TAM_3_ (0.3 mL 1 L^−1^ of water culture).

Cyclopoida copepods *O. nana* have been recognized as the optimal species for large-scale production^81,82^. However, the development of cost-effective protocols is necessary to further enhance the production process^53,83^. Generally, copepod mass production heavily relies on microalgae species and/or dry feedstuffs, and encounters various challenges in culture, including low yields, long generation time, slower population growth, increased time requirements for mass production, and the high costs of large-scale production^41,83^. To address these issues, ongoing efforts are focused on developing feed additive strategies that can optimize copepod production, improve yields, reduce generation time, minimize seasonal variations, and lower overall costs. These feed additive strategies should be highly practical, economically viable, and have the potential to serve as alternative diets, improve the quality of dry feedstuffs, and replace microalgae while maximizing the reproductive capacity and population of Cyclopoida copepods, specifically *O. nana*^84^. Table 4 shows the fatty acids profile (%) of *O. nana* feed starch supplemented with different levels of TAM®, after 21 days of experiment.

**Table 4:** Fatty acids profile (%) of copepod *Oithona nana* feed starch supplemented with different levels of TAM.

Fatty acids profile	Treatments				
	CS	S-TAM_1_	S-TAM_2_	S-TAM_3_	S-TAM_4_
C8:0	n.d	2.3	n.d	2.4	1.04
C12:0	1.53	2.59	20.32	12.01	22.82
C14:0	0.49	5.54	4.2	4.67	2.56
C15:0	2.83	0.31	2.72	1.2	0.85
C16:0	17.77	33.43	25.57	38.5	33.17
C16:1	3.31	2.98	7.61	2.09	2.45
C17:0	0.42	0.81	0.86	2.13	2.42
C17:1	n.d	0.41	n.d	n.d	0.95
C18:0	3.13	6.62	6.67	6.65	5.99
C18:1c ω9	10.2	14.94	11.37	12.86	13.26
C18:1t ω9	1.17	1.87	3.94	2.78	2.49
C18:2c ω6	13.1	4.34	3.2	1.69	2.41
C18:3ω3	2.89	0	0	0	0.58
C20:0	1.55	0.62	0	0	0
TFA (%)	62.39	76.76	86.46	83.98	90.99
SFT (%)	24.59	46.01	53.67	60.91	63.81
Omega-3 (%)	2.89	0	0	0	0.58
Omega-6 (%)	13.1	4.34	3.2	1.69	2.41
Omega9 (%)	11.37	16.81	15.31	15.64	15.75
MUFA (%)	14.68	20.2	22.92	17.73	19.15
PUFA (%)	15.99	4.34	3.2	1.69	2.99
HHR	1.32	0.46	0.29	0.26	0.28

n.d.: Not detected. TFA: total fatty acids (%), SFT: saturated fatty acids (%), MUFA: monounsaturated fatty acids (%), PUFA: polyunsaturated fatty acids (%), and HHR: Hypocholesterolemic/Hypercholesterolemic ratio.

Table 4 shows that compared to the control group, the copepod that fed starch supplemented with different levels of TAM® achieved the highest TFA, SFA, MUFA, and Omega9 content (%). The highest percentages of C12:0, C14:0, C16:0, C17:0, C18:0, C18:1c ω9, and C18:1t ω9 were reported in TAM® groups. These findings may be attributed to the phytochemical compounds and the nutritional value of TAM® which contains nine phytochemical compounds, one of them is oleic acid (fatty acid) and three of them are fatty acid methyl esters compounds; (1) Tridecanoic acid methyl ester, (2) 9,12-Octadecadienoic acid methyl ester, and (3) γ-Linolenic acid methyl ester^77^. While compared to TAM® groups, the copepod fed the control group achieved the highest Omega-3, Omega-6, PUFA content (%), and HHR ratio. In all experimented groups, the absence of the highly unsaturated fatty acids, especially EPA and DHA, maybe because the *O. nana* individuals were fed only corn starch, during the 15-day acclimatization period, with a concentration of 1 g 10^−6^ individuals, to ensure that the digestive tract of *O. nana* was completely emptied from any other food before the beginning of the experiment.

### Chromium remediation

#### Plackett–Burman design screening of important variables influencing the TAM® biomass’s percentage of Cr^6^^+^ elimination

Adsorption of Cr^6+^ ions from aqueous solutions is a technology that shows promise for treating wastewater. Its foundation lies in natural materials’ capacity to absorb Cr^6+^ ions from wastewater by metabolically mediated absorption pathways or physicochemical adsorption. Using the Plackett–Burman Design (PBD) statistical analysis method, the impact of the six factors examined in this study namely, Cr^6+^ concentrations, static/agitation, biomass concentrations, temperature, pH, and contact time on Cr^6+^ removal percentage was determined. pH is the most significant process parameter that affects the site’s availability to the sorbate as protons and metal cations fight for binding sites since it was discovered that ion exchange is the primary mechanism of adsorption^85^. Table 1 displays the Plackett–Burman design matrix that was utilized to identify the key factors influencing the percentage of Cr^6+^ removal from aqueous solutions by TAM® biomass. There were 12 runs of the experiment. Table 1 displays the percentage of Cr^6+^ removal in each run along with the levels of coded and actual values for the evaluated independent variable. The data indicated a range in Cr^6+^ removal % among the 12 trials, ranging from 52.68 to 93.65%. This variation indicated that to achieve maximal chromium removal efficiency needed to be improved by process modification. Results indicated that run no. 6 had the highest chromium elimination percentage (93.65%). Using a Plackett–Burman design, the link between the independent variables and the percentage of chromium removal was examined for their effects on the percentage of chromium removal. Each factor’s coefficient indicates how much of an impact it has on the elimination of chromium. Analysis of the regression coefficients of the six factors (Table 5) showed that chromium ions concentration, agitation-static, and temperature with coefficient values (3.2, 1.6, and 3.5, respectively) had positive effects on chromium removal % which means that the increase in chromium ions concentration, agitation, and temperature factors could exert a positive effect on chromium removal.

**Table 5:** Analysis of variance (ANOVA) and regression statistics for the Plackett–Burman experimental data for TAM biomass-mediated Cr^6+^ elimination.

Source	Symbol	df	Coefficient	*t-*Stat	*P*-value		
			94.09	4.00	0.013		
Contact time (min.)	A	1	− 5.34	− 0.08	0.04		
Cr concentration (mg L^−1^)	B	1	3.26	0.27	0.80		
pH	C	1	− 0.84	− 0.08	0.94		
Temperature (°C)	D	1	3.52	− 0.90	0.04		
Biomass dosage (g)	E	1	− 0.19	− 0.27	0.80		
Static-Agitation	F	1	1.66	0.85	0.04		
R-Sq(adj)	0.885						
R^2^	0.940						

	df	SS	MS	F	Significance F	Residual error	Total
Regression	6	67.425	79.22	39.45	0.935	5	11

Also, Table 5 presents the results of the Analysis of Variance (ANOVA) and regression statistics for the Plackett–Burman experimental design in the process of Cr^6+^ (hexavalent chromium) removal using TAM biomass. This analysis evaluates the impact of various factors on the percentage of chromium removal and assesses the statistical significance of these factors. The results of contact Time (A) showed that the coefficient is − 5.34. This indicates that increasing the contact time negatively affects the chromium removal percentage^86^. The reason may be reaching an equilibrium state where no further adsorption occurs, and extended contact might even lead to desorption of the previously adsorbed chromium. While the t-Stat: is − 0.08 and the P-value: is 0.04. The low t-stat suggests a slight effect, but the P-value (less than 0.05) indicates statistical significance, meaning the impact of contact time is indeed relevant. The results of Chromium Concentration (B) showed that the coefficient (3.26) which has a positive effect suggests that increasing the chromium concentration enhances removal efficiency, likely due to the increased driving force for adsorption. The values of t-Stat: 0.27 and P-value: 0.80, the low t-Stat and high P-value indicate that this effect is not statistically significant, implying that chromium concentration’s impact is not strong in this experiment. The results of pH (C) showed that the coefficient (− 0.84) indicates that a lower pH negatively affects chromium removal due to the competition between protons and chromium ions for the adsorption sites. The high P-value (0.94) shows no statistical significance, suggesting that pH did not have a strong impact on chromium removal under the experimental conditions. The results of Temperature (D) showed that the coefficient (3.52). A positive effect on chromium removal indicates that higher temperatures may improve the adsorption rate by increasing the kinetic energy of the ions. Although the t-Stat (− 0.90) is negative, the P-value below 0.05 indicates that temperature has a statistically significant effect. Biomass Dosage (E) coefficient = − 0.19 the value of a negative effect indicates that increasing biomass dosage does not proportionally improve removal efficiency. This could be due to the agglomeration of biomass particles, reducing the effective surface area available for adsorption^87^. While t-Stat: − 0.27 and P-value: 0.80, these values indicate that the effect of biomass dosage is not statistically significant. Static-Agitation (F) coefficient is 1.66. The positive impact suggests that agitation improves chromium removal efficiency by facilitating contact between the ions and adsorption sites. The P-value below 0.05 indicates that the effect of agitation is statistically significant^88^.

*Model quality indicators:* The values of R^2^ = 0.940 and R^2^(adj) = 0.885 suggest that the model explains 94% of the variability in the data, indicating a high-quality fit for the factors studied. F-value = 39.45 and Significance F = 0.935, these values showed that the high F-value demonstrates a strong significance of the model, suggesting that the factors have a meaningful impact on the chromium removal process.

**Fig. 7:**
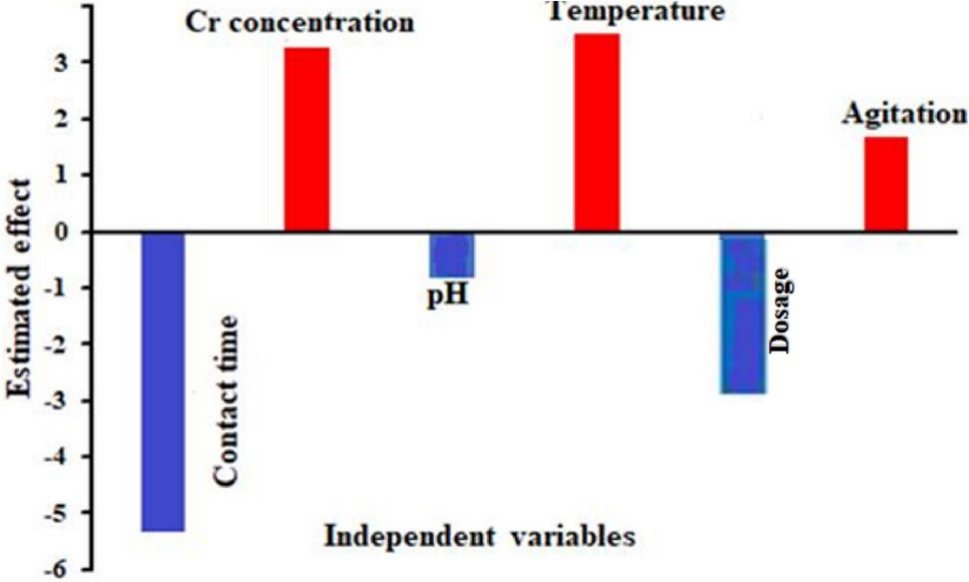
The estimated effects of independent factors on Cr^6+^ removal by TAM biomass using the Plackett–Burman design.

On the other hand, Tahir et al.^89^ found that agitation improves adsorption and makes it easier for the metal ions in solution to make appropriate contact with the biomass-binding sites, which in turn encourages the efficient transport of adsorbate ions to the TAM® adsorbent sites. Where, contact time, pH, and biomass dosage with coefficient values (− 10.6, − 1.6, and − 0.37; respectively) had negative effects which mean that the decrease in pH, contact time, biomass amount, and agitation/static levels could exert a positive effect on chromium ions removal (Fig. 7).

Figure 7 displays the estimated effects of different independent variables on the percentage of Cr^6+^ removal by TAM® biomass, based on the Plackett–Burman design. The figure shows both positive and negative impacts of the variables on chromium removal efficiency, providing insights into which factors contribute most significantly to the process. The positive effect indicates that increasing the concentration of Cr^6+^ in the solution enhances the removal efficiency. Higher concentrations likely increase the driving force for adsorption, leading to better chromium uptake by the biomass^90^. Similarly, the positive effect of temperature suggests that higher temperatures improve chromium removal. This may be due to increased molecular movement, which facilitates better interaction between Cr^6+^ ions and the biomass^91^ In addition, the positive effect of agitation implies that stirring or mixing the solution improves the contact between Cr^6+^ ions and the TAM® biomass, promoting more efficient adsorption. Nonetheless, The negative effect indicates that longer contact time does not necessarily improve Cr^6+^ removal. This might be because, after a certain point, the adsorption sites are saturated, and extended contact can even lead to desorption^92^. The negative effect of pH suggests that lower pH values (more acidic conditions) hinder Cr^6+^ removal. This could be due to increased competition between protons (H^+^) and Cr^6+^ ions for the available adsorption sites. In addition, the negative effect of increasing biomass dosage indicates that adding more biomass does not linearly increase removal efficiency. This may occur due to particle agglomeration, which reduces the effective surface area for adsorption^93^.

**Fig. 8:**
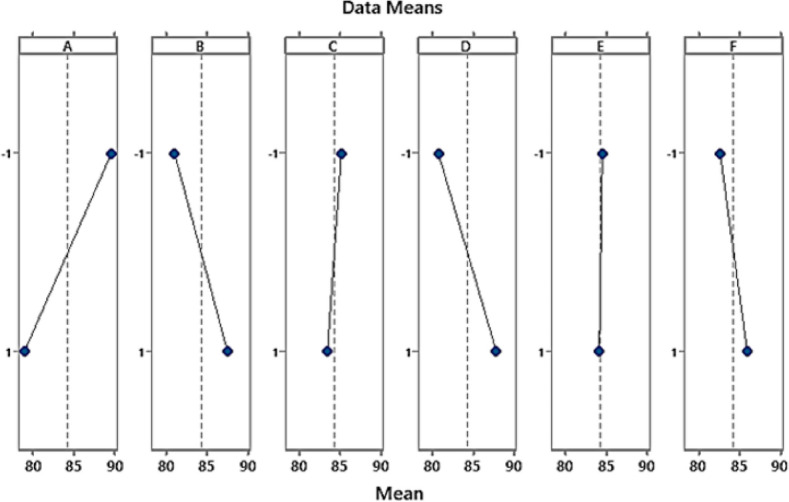
Main effects plot for percentage removal of Cr^6+^ by TAM (**A**: Contact time (min.); **B**: Cr concentration (mg L^−1^); **C**: pH; **D**: Temperature (°C); **E**: Biomass dosage (g); **F**: Static-Agitation).

The estimated impact of each factor on the percentage removal of chromium ions is displayed in Table 5 and Fig. 8. While a near-zero effect suggests that a variable has little to no effect, a substantial effect, whether positive or negative, shows that the parameter has a significant impact on the elimination of chromium ions. Figure 8 is a main effects plot showing the impact of six independent factors (A: Contact time, B: Chromium concentration, C: pH, D: Temperature, E: Biomass dosage, and F: Static-Agitation) on the percentage of Cr^6+^ removal by TAM® biomass. This plot helps to visually assess how changes in the levels of each factor (from low to high) influence the mean percentage of chromium removal. The plot for contact time shows a rising trend, indicating that increasing contact time improves Cr^6+^ removal efficiency. This suggests that allowing more time for the adsorption process enhances chromium uptake, possibly up to a certain point^94^. The increasing slope indicates that higher chromium concentrations positively impact the removal efficiency. This is likely because a higher concentration gradient drives more chromium ions toward the available adsorption sites on the biomass. While, the plot shows a downward trend with higher pH levels, suggesting that Cr^6+^ removal efficiency decreases at higher pH. At lower pH, there may be more competition between H^+^ ions and Cr^6+^ ions for adsorption sites, reducing removal efficiency. The decreasing slope in temperature indicates that higher temperatures negatively affect chromium removal. This could be due to the desorption of Cr^6+^ ions at elevated temperatures or changes in the adsorption site characteristics^95^. The line shows a flat trend, indicating that changes in biomass dosage (E) have minimal impact on chromium removal. This suggests that increasing the amount of biomass beyond a certain level does not necessarily improve the efficiency of the removal process. Besides, the decreasing slope shows that the agitation process helps improve Cr^6+^ removal. With agitation, the mass transfer rate increases, allowing more chromium ions to reach the adsorption sites on the biomass^96^.

The findings showed that temperature and high initial chromium ion concentrations had a favorable impact on the percentage of chromium ions eliminated. On the other hand, high amounts of the other variables as pH, contact time, and adsorbent amount had a detrimental impact on the percentage of chromium ions removed. The amount of biomass, the period of contact, and the agitation/static are three less significant factors. While the fits and diagnostic for unusual observation was run number 11, with a percentage removal of 52.6%. A particularly effective tool for illustrating the relative significance of parts is the Pareto chart. It has bars, which are used to indicate individual numbers in descending order.

**Fig. 9:**
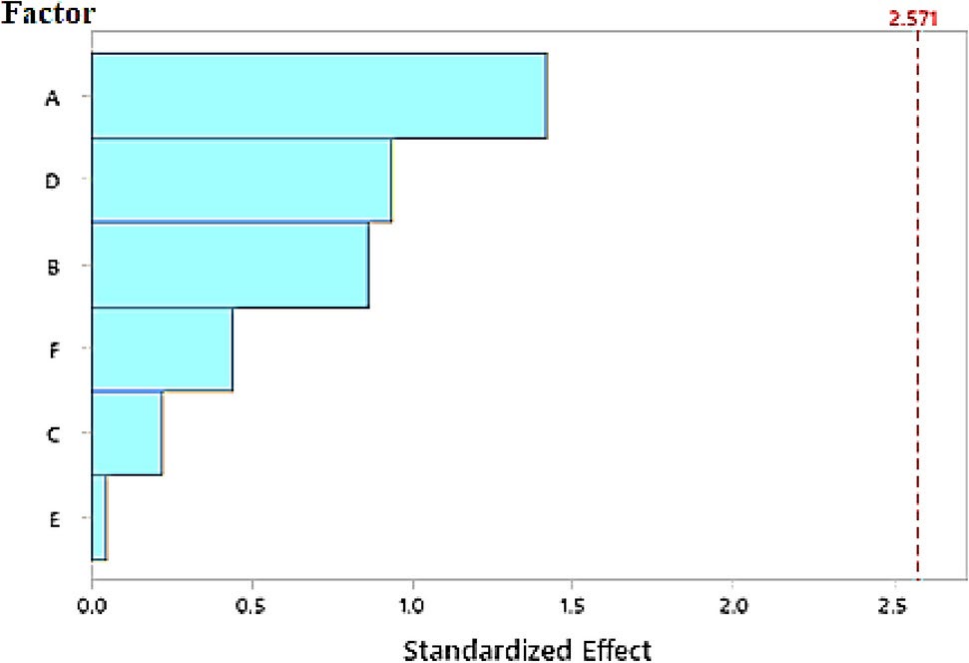
Pareto chart of standardized effect on Cr^6+^ removal by TAM using Plackett–Burman design (response is percentage removal, α = 0.05). (**A**: Contact time (min.); **B**: Cr concentration (mg L^−1^); **C**: pH; **D**: Temperature (°C); **E**: Biomass dosage (g); **F**: Static-Agitation).

The Pareto chart is a useful tool for visualizing which factors have the most significant impact on the response variable (Cr^6+^ removal), and it helps to prioritize factors for further optimization^97^. Each bar in the chart represents a factor affecting Cr^6+^ removal, with the length of the bar indicating the magnitude of the standardized effect. The factors are listed in descending order of their impact, with "A" (Contact time) being the most significant, followed by "D" (Temperature), "B" (Cr concentration), "F" (Static-Agitation), "C" (pH), and "E" (Biomass dosage) as presented in Fig. 9. The vertical red dashed line at 2.571 represents the critical value for statistical significance at α = 0.05. Factors with bars that extend beyond this line are considered statistically significant, meaning they have a meaningful effect on Cr^6+^ removal. Contact Time (A) shows the highest effect, suggesting that varying the contact time has the most considerable impact on Cr^6+^ removal efficiency. However, it did not cross the threshold for statistical significance, indicating the need for refining the experimental design or increasing the number of trials to improve the power of detecting a significant effect^98^. In addition, Temperature (D) and Cr Concentration (B) also have relatively high effects, implying that these factors influence the adsorption process. Elevated temperatures may enhance the adsorption rate, while higher Cr concentrations create a stronger driving force for mass transfer to the adsorption sites. Static agitation (F) and pH (C) exhibit moderate effects, indicating that mixing and pH adjustment play a role in optimizing the adsorption process. Agitation helps in the distribution of Cr^6+^ ions for better contact with the biomass, while pH may influence the ionization state of chromium and the surface charge of the biomass^99^. Finally, the biomass dosage (E) has the least effect on the Cr^6+^ removal efficiency, suggesting that adding more biomass beyond a certain point does not significantly enhance the adsorption capacity.

### The plot of normal probability (NPP)

**Fig. 10:**
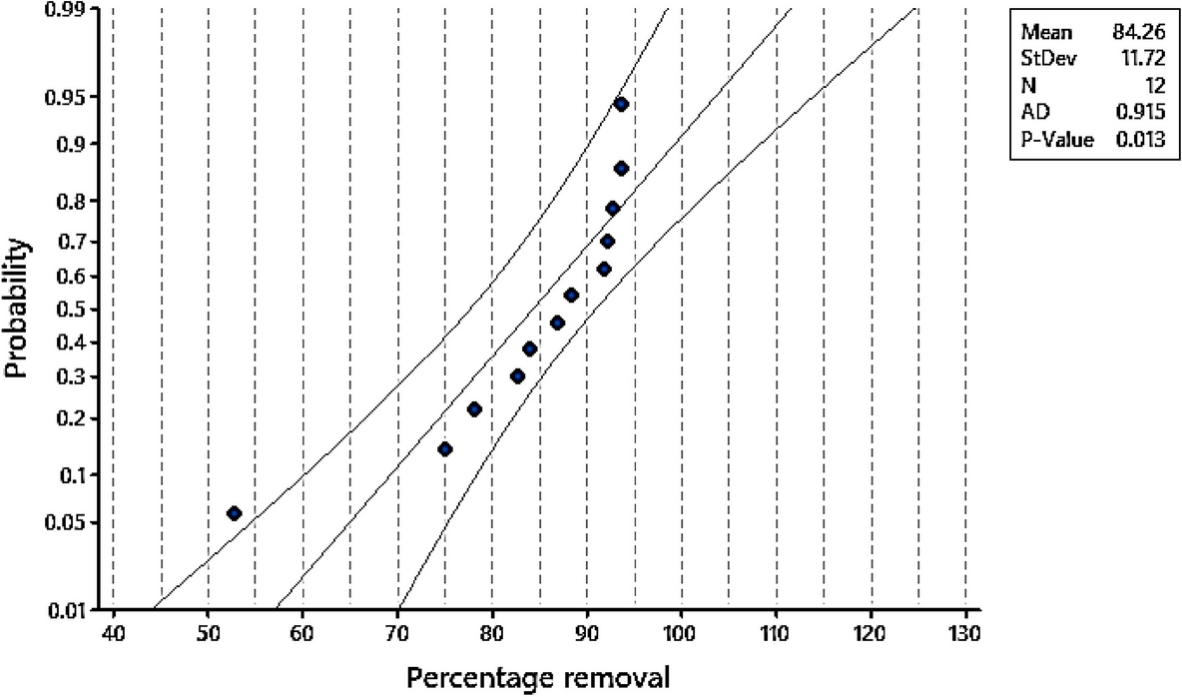
Probability plot of percentage removal (normal − 95%).

The residuals’ normal probability plot (NPP), plotted against the model’s expected outcomes, is displayed in Fig. 10. The data points for the residuals from the fitted model for the Cr^6+^ removal percentage are located along the horizontal line; yet, the data appear to be regularly distributed and provide information about the mathematical equations’ accuracy. The model was shown to be significant by the ANOVA of the Plackett–Burman design, as seen by the t-Stat (0.541) and the Fisher’s F-test (0.935), which both had extreme P-values (0.093). A variable is deemed important when its confidence level is more than 95%. As seen in the obtained results, the t-values and P-values were calculated for each independent variable and were a useful tool for determining the significance of each parameter. The fit of the model was checked by the determination of the coefficient (R^2^). The R^2^ value is always between 0 and 1. If the value of R^2^ is close to 1, the model is stronger and better to predict the response.

In the present study, the R^2^ value is 0.940 indicating that up to 94.0% variability in Cr removal % could be calculated by the model. In addition, the value of the adjusted determination coefficient (adjusted R^2^ = 0.885), shows the significance of the model (Table 5). Therefore, R^2^ and R^2^ adj emphasized that the model is significant and suitable to explain the relationship between the selected variables and the Cr^6+^ removal %. However, the predicted R^2^ value obtained is 0.8701, indicating that the model does not explain only 13% of the total variations. By applying multiple regression analysis on the experimental data, the experimental results of the Plackett–Burman design were fitted with the regression equation in uncoded units’ equation which represents the Cr^6+^ removal % as a function of the pH, temperature, contact time, Cr^6+^ concentration, biomass dosage and agitation–static.

8 $${\text{Percentage removal of Cr}}^{{{6} + }} = {84}.{26} - {5}.{\text{34A}} + {3}.{\text{26B}} - 0.{\text{84C}} + {3}.{\text{52D}} - 0.{\text{19E}} + {1}.{\text{66F}}$$

In a confirmatory experiment, to evaluate the accuracy of Plackett–Burman, the conditions expected to be optimum for maximum Cr^6+^ removal by TAM® from aqueous solutions were contact time of 60 min, initial Cr^6+^ concentration of 60 mg L^−1^, pH 3, temperature 35°C, and TAM® biomass of 0.05 g at agitation condition. Under these conditions, the maximum removal percentage of Cr^6+^ was 93.65%. In the present study, the metal concentration (mg L^−1^) and pH are non-significant factors (*P*-value = 0.799 and P-value = 0.941).

**Fig. 11:**
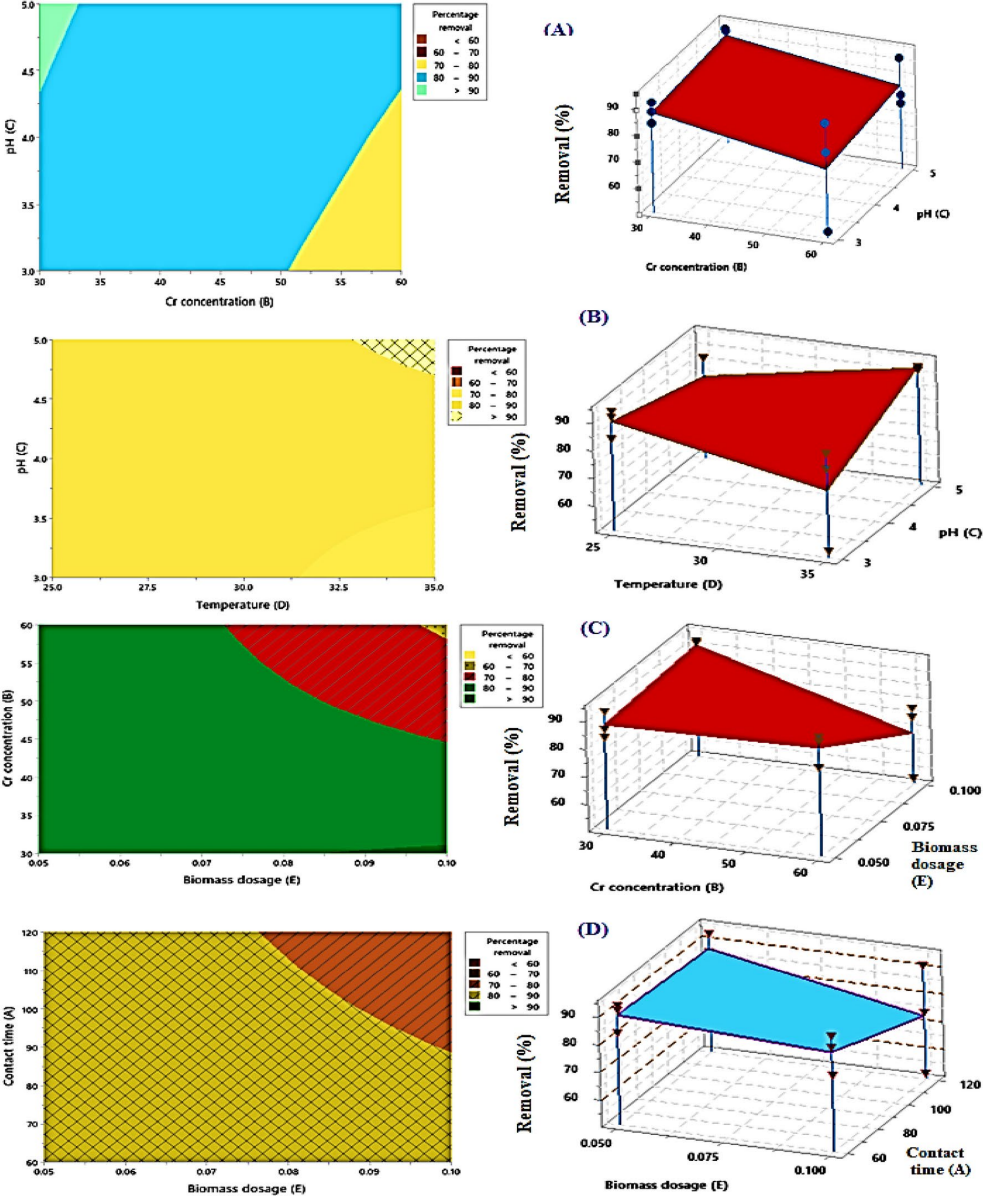
shows the 3D and contour plots of the effects of temperature (X3), pH (X1), and Cr^6+^ concentration (X2), which affect the removal of Cr^6+^ by TAM biomass dosage (X4). (**A**: Contact time (min.); **B**: Cr concentration (mg L^−1^); **C**: pH; **D**: Temperature (°C); **E**: Biomass dosage (g); **F**: Static-Agitation).

Figure 11 shows the 3D and contour plots of the effects of pH (X1), Cr^6+^ concentration (X2), and temperature (X3), as well as how these factors interact to affect the removal of Cr^6+^ by TAM® biomass dosage (X4). As the original pH decreased, Fig. 11A illustrates how, up to a particular pH value, the Cr^6+^ removal % increased.

However, the percentage of Cr^6+^ removed rose as the concentration of Cr^6+^ decreased, and higher concentration levels of Cr^6+^ support comparatively high percentages of Cr^6+^ removal above 80%. The highest percentage of 84% for the removal of Cr^6+^ at the optimal levels of pH and Cr^6+^ concentration, which are 3 and 60 mg L^−1^, respectively. The efficiency of Cr^6+^ removal as a function of starting pH and temperature is displayed in the 11B surface plot and contour plot that corresponds to it in Fig. 11B.

The results provide evidence that Cr^6+^ removal increased at pH values higher than 3, after which it dropped. A comparatively high percentage of Cr^6+^ removal is supported by both lower and higher temperature levels, with the maximum percentage of Cr^6+^ removal being located in the temperature center. At the optimal anticipated temperatures of 25 °C and pH of 3, respectively. The effects of biomass dosage and Cr^6+^ concentration on the effectiveness of Cr^6+^ removal are displayed in the matching contour plot (Fig. 11C) and C plot. The highest removal of Cr^6+^ metal was reached at low levels of initial Cr^6+^ concentration and adsorbent dose of 60 mg L^−1^ and 0.05 g, respectively. The nature of the adsorbent and the number of binding sites (the concentration and kind of the amount of biomass) are other significant factors in adsorption. An increase in the initial Cr^6+^ concentration increases the amount of absorbed Cr^6+^ per unit weight of the biomass but decreases removal efficiency. An increase in temperature typically improves Cr^6+^ removal by increasing the adsorbate’s surface activity and kinetic energy^100^.

Future research on TAM® will address recent advanced technologies like nanoparticles and nanoencapsulation, to (1) enhance TAM® potential applications in different fields, (2) ensure the delivery of bioactive compounds and microagricultural integration for optimized plant growth, (3) increase the efficiency of aquafeed for aquatic animals, and (4) exploring advanced bioremediation strategies using TAM for pollutant removal is essential. Focusing on these areas can fully realize the potential of seaweed extracts, particularly TAM®, benefiting industries such as aquafeed, plant growth stimulation, and environmental remediation. This comprehensive approach will advance technology and promote the environment through sustainable seaweed extract utilization. On the other hand, future research on TAM® will focus on bioactivity studies, environmental impact assessments, and innovative applications in pharmaceuticals and cosmetics. Moreover, future research will also address the economic considerations of TAM®, involving cost–benefit analyses, market demand studies, and supply chain optimization for scalability and sustainability.

## Conclusion

Seaweed extracts offer significant ecological and economic benefits through their diverse species and unique bioactive compositions. Liquid seaweed extracts, like TAM®, present a sustainable solution with broad applications. The study demonstrated the effective removal of Chromium (Cr^6+^) ions from aqueous solutions using the seaweed liquid extract (TAM®). The optimized process achieved a maximum removal percentage of 93.65% using specific conditions for pH, temperature, contact time, and TAM® biomass dosage. TAM® at a level of 0.037 mg mL^−1^ showed high root length enhancement on *V. faba* (184%) and *T. foenum-graecum* (188%). The copepod *O. nana* fed on starch supplemented with TAM® at a level of 0.3 mL L^−1^, compared to the control group that fed starch only, showed the highest increment in population growth (134.74%), fecundity (270.16%), and population composition of males (133.45%), adults (120.37%), and nauplius (203.18%). Moreover, achieved the highest Omega-9 content. In conclusion, it is shown that TAM® is a feasible, efficient, and sustainable solution for biodegradable adsorbent for the Cr^6+^ from aqueous solution, enhances plant seed germination and root length*,* and is a novel feed additive for marine copepod *O. nana*, especially in marine invertebrate hatcheries. Further exploration of TAM® potential is crucial for progress in research and technology. Ongoing innovation in aquafeed, plant growth stimulation, and environmental remediation will benefit from sustainable seaweed extract use. Understanding the versatility of seaweed extracts promotes a harmonious coexistence between humans and the environment. The study highlights TAM® as a novel, sustainable solution for biofertilization, bioremediation, and enhancing marine copepod O. nana growth in marine hatcheries.

## Supplementary Information

Supplementary Information.

## Data Availability

The datasets used and/or analyzed during the current study are available from the corresponding author upon reasonable request.
