# Supplementary material for: Seaweed liquid extract AS novel sustainable solutions for phycobioremediation plant germination, and feed additive for marine invertebrate copepod
Source: Sci Rep. 2024 Nov 28;14:29553. doi: 10.1038/s41598-024-80389-z (PMC11605070; doi:10.1038/s41598-024-80389-z)
Supplement: Supplementary file 1 — Supplementary Information. [file 41598_2024_80389_MOESM1_ESM.doc]

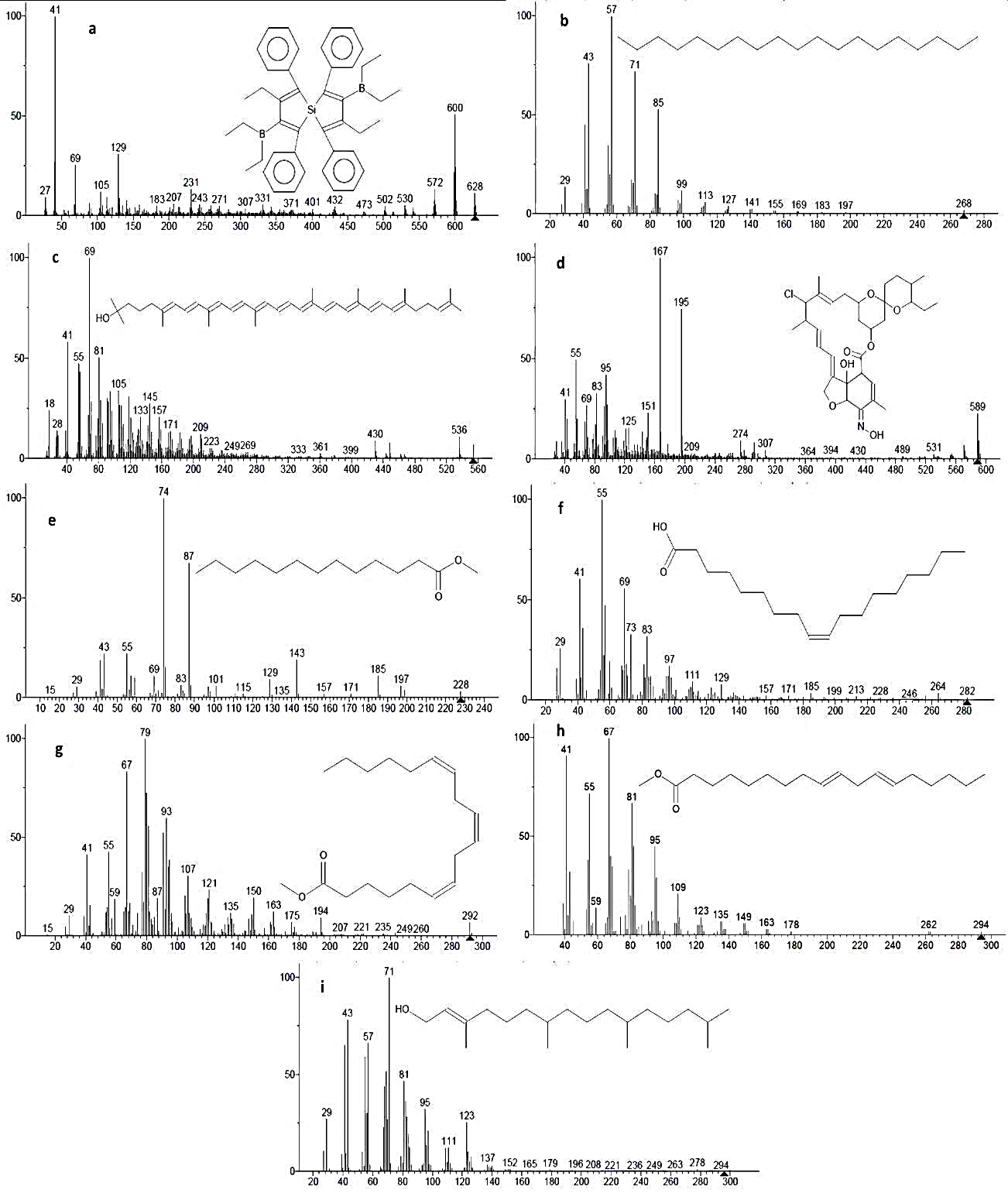


**Figure 1.** Mass spectra and retention times of nine phytochemical compounds in TAM®. (**a**) 5-Silaspiro[4.4]nona-1,3,6,8-tetraene,3,8-bis(diethylboryl)-2,7-diethyl-1,4,6,9-tetraphenyl- (8.99 min); (**b**) nonadecane (16.31 min); (**c**) rhodopin (19.45 min); (**d**) milbemycin B (20.07 min); (**e**) tridecanoic acid methyl ester (20.90 min); (**f**) oleic acid (21.63 min); (**g**) γ-linolenic acid methyl ester (23.74 min); (**h**) 9,12-octadecadienoic acid, methyl ester, (E, E)- (24.02 min); and (**i**) phytol (24.37 min). Cited from our previous published work[^1^](#_ENREF_1)

Reference

1 Ashour, M., Mabrouk, M. M., Abo-Taleb, H. A., Sharawy, Z. Z., Ayoub, H. F., Van Doan, H., Davies, S. J., El-Haroun, E. & Goda, A. M. S. A. A liquid seaweed extract (T^A^M®) improves aqueous rearing environment, diversity of zooplankton community, whilst enhancing growth and immune response of Nile tilapia*, Oreochromis niloticu*s, challenged b*y Aeromonas hydrophi*la*. Aquacultu*r**e 5**43, 736915, doi:10.1016/j.aquaculture.2021.736915 (2021).
